# Hand hygiene compliance and associated factors among health care providers in Central Gondar zone public primary hospitals, Northwest Ethiopia

**DOI:** 10.1186/s13756-019-0634-z

**Published:** 2019-11-26

**Authors:** Garedew Tadege Engdaw, Mulat Gebrehiwot, Zewudu Andualem

**Affiliations:** Department of Environmental and Occupational Health and Safety, Institute of Public Health College of Medicine and Health Sciences, University of Gondar, Gondar, Ethiopia

**Keywords:** Hand hygiene compliance, Health care provider, Public primary hospitals

## Abstract

**Background:**

Poor hand hygiene compliance is one in all the leading contributory factors to healthcare-associated infections. This is an important source of complications across the continuum of care and poses a serious threat to people admitted to hospitals. However, the magnitude and associated factors of hand hygiene compliance in public primary hospitals were not well investigated in Ethiopia. Therefore, this study was conducted to assess hand hygiene compliance and associated factors among health care providers in Central Gondar Zone public primary hospitals, Northwest Ethiopia.

**Methods:**

An Institutional based cross-sectional study was conducted from March to April 2019 among health care providers. The data were collected using self-administered questionnaires and observational checklists. Using Epi Info 3.1, data was entered and analyzed using SPSS version 23. The data were analyzed using descriptive statistics and logistic regression models. A *p*-value less than 0.05 with 95% confidence interval was used to declare statistical significance**.**

**Results:**

Of 335 study participants, 50 (14.9%), had good hand hygiene compliance. Training on hand hygiene (AOR = 8.07, 95%CI: 2.91, 22.39), availability of adequate soap and water for hand hygiene (AOR = 5.10, 95%CI: 1.93, 13.52), availability of alcohol-based hand rub (AOR = 3.23, 95%CI: 1.32, 7.92), knowledge about hand hygiene (AOR = 6.74, 95%CI: 2.96, 15.34) and attitude towards hand hygiene (AOR = 2.15, 95%CI: 1.04, 4.46) were factors associated with hand hygiene compliance.

**Conclusion:**

The overall level of hand hygiene compliance among health care providers was poor. Training, availability of adequate soap and water, availability of alcohol-based hand rub, knowledge on hand hygiene, and attitude of health care providers were significantly associated with hand hygiene compliance.

## Background

Hand hygiene is the compliance of cleansing hands with soap and water or with antiseptic hand rub to remove transient microorganisms from hands and maintain the condition of the skin [1]. It is one of the most important health issues in the world and could be a single efficient and sensible life to reduce the incidence of health-related infections and thereby reveal antimicrobial resistance in all settings, from advanced health care systems to primary health care [2].

Performing hand hygiene activities within the health institutions by health care providers with Alcohol-Based Hand Rub (ABHR) at multiple times causes them to feel uncomfortable. The right technique and duration of handwashing using soap and water and ABHR are very important to confirm the removal of microorganisms. This will be done before and once touching the patient, before handling an invasive device for patient care, once contact with body fluids or excretions, mucous membranes, non-intact skin, or wound dressings, moving from a contaminated body web (site) to a different body site throughout care of the identical patient [3, 4].

Healthcare-associated infections (HCAIs) have an excellent impact on morbidity, length of hospital stays, and treatment prices [5]. Health care providers’ hands are the main usual mode of the vehicle for the transmission of HCAIs. About 50% of HCAIs happens due to the hand of health care providers [6].

During patient care, unless there are recommended hand hygiene compliance of health care suppliers uninterrupted, hands are contaminated with a microorganism [7]. Annually regarding many numerous patients have suffered from HCAIs worldwide [8]. Improper hand hygiene by HCPs is answerable for regarding 40% of health facility infections [9]. This infection is answerable for nearly 50% of the deaths that occur on the far side a pair of weeks of age [3].

Therefore, the aim of this study was to assess hand hygiene compliance and associated factors among health care providers in Central Gondar Zone public primary hospitals, Northwest Ethiopia, 2019.

## Methods and materials

### Study design, period, and study area

The institutional-based cross-sectional study was conducted to assess hand hygiene compliance and associated factors among health care providers in central Gondar zone public primary Hospitals, northwest Ethiopia, from March to April, 2019.

The study was conducted in the region of northwest Amhara in five primary hospitals named, Kolladiba, Aykel, Wogera, Delgi, and Guhala. These hospitals have clinical and administrative staff. The number of health care providers differ from hospital to hospital. Kolladiba (70), Aykel (80), Wogera (66), Delgi (54) and Guhala (71) public primary hospitals.

### Eligibility criteria

#### Inclusion criteria

All health care providers who had worked at least 6 months in the central Gondar zone public primary hospitals who were included in this study.

#### Exclusion criteria

Health care providers who were not present during the data collection time due to different reasons were excluded.

### Sample size determination

The required sample was calculated using single population proportion formula with the assumption of the proportion *P*-value is 50% (there is no previous study), and marginal error 5%, and a standard Z score of 1.96 corresponding to 95% confidence interval, by adding 5% non-response rate the final sample size was 403. But in the study setting, the total number of healthcare providers was 341. Due to a small number and affordable to do research on all health care providers, we have included all HCP from five public primary hospitals.

### Data collection tools

Data were collected by a self-administered questionnaire and observation checklist adapted from different literature [6, 10, 11]. The questionnaire had questions related to socio-demographic, knowledge, attitude, and practice. This questionnaire had both open and close-ended questions. The English version of the questionnaire was translated into the local language (Amharic) and it was translated back into English by the third person to check its consistency. Two days training was given on the data collection tools, questioning techniques, and ethical issues, interview techniques, ways of obtaining the verbal consent and how to interact with respondents as precautions for data collectors and supervisors. The data collectors were Nurses and Environmental health professionals under the supervision of two field supervisors.

### Operational definitions

#### Good hand hygiene compliance

Health care providers who practiced all of the hand hygiene moments from the observational checklist [12].

#### Poor hand hygiene compliance

Health care providers who do not practice at least one of the hand hygiene moments from the observational checklist [12].

### Data processing and analysis

For completeness and consistency, the collected data were rechecked. Data were entered in Epi-Info version 3.1 software and exported to SPSS version 23 for further analysis. Descriptive statistics was employed for the socio-demographic characteristics of the respondents. Bivariable and multivariable logistic regressions were carried out to identify significantly associated variables with hand hygiene compliance by backward logistic regression variable selection method. Crude Odds Ratio, and Adjusted Odds Ratio (AOR) with 95%CI were computed to determine the associated factor of hand hygiene compliance and P- value less 0.05 was considered as declared statistically significant. Hosmer and Lemeshow goodness of fit (*P* > 0.05) were used to test the fitness of the model during analysis.

## Results

### Socio-demographic characteristics of respondents

A total of 341 respondents were aimed for this study. Of these, 335 respondents were recorded with a response rate of 98.2%. The mean age of the respondents was 28 ± 5.6 (± SD) years. Most of the 299 (89.3%) study respondents were Orthodox Christians in religion. Professional respondents were 120 (35.8%) Nurses **(**Table 1**)**.

**Table 1:** Socio-demographic characteristics of the health care providers Central Gondar Zone public primary hospitals, Northwest Ethiopia, 2019 (*n* = 335)

Variable		Frequency	Percent (%)
Age	18–24	55	16.4
	25–34	243	72.5
	> 35	37	11.1
Sex	Male	238	71
	Female	97	
Religion	Orthodox	299	89.3
	Muslim^a^	36	10.7
Profession	Physician	41	12.2
	Nurse	120	35.8
	Laboratory	34	10.1
	Midwives	74	22.1
	Others^b^	66	19.7
Level of education	Diploma	87	26
	Bachelor	200	59.7
	2nd degree and above^c^	48	14.3
Marital status	Married	159	47.5
	Single^d^	176	52.5
Working experience	0–5	201	60
	6–10	63	18.8
	11–15	36	10.7
	>16	35	10.5
Unit of work	OPD	74	22.1
	Emergency	36	10.7
	Inpatient	53	15.8
	Laboratory	34	10.1
	OR	41	12.2
	GYN Obs	63	18.8
	Others^e^	34	10.1

^a^Protestant, Others

^b^HO, Radiography, Anaesthesia, Optometry, pharmacy

^c^Masters and GP

^d^Divorced, Widowed,

^e^Triage

### Reasons for not practicing hand hygiene

**Fig. 1:**
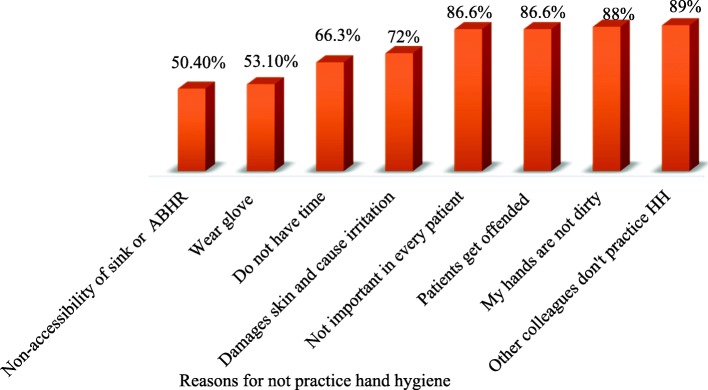
Study participants’ reasons for not practicing hand hygiene in Central Gondar Zone public primary hospitals, Northwest Ethiopia, 2019

Half of the study participants argue that the inaccessibility of sink and ABHR is a reason for not practicing good hand hygiene **(**Fig. 1**)**.

### Hand hygiene compliance of health care providers

In this study, the prevalence of hand hygiene compliance from observation was 14.9%, (95% CI: 11.3, 18.5). Above half 181 (54%) of the respondents were knowledgeable about hand hygiene compliance. Of the respondents, 140 (41.8%) health care providers are trained about hand hygiene compliance and 177 (52.8%) assured the presence of alcohol-based hand rub in their working area (Table 2).

**Table 2:** Variables related to hand hygiene compliance of health care providers in Central Gondar Zone public primary hospitals, Northwest Ethiopia, 2019 (*n* = 335)

Variables		Frequency	Percent (%)
Knowledge	Good	181	54
	Poor	154	46
Frequently keep hand hygiene	Yes	301	89.9
	No	34	10.1
Taking training	Yes	140	41.8
	No	195	58.2
Hospital promotes the importance of HH to the staffs	Yes	161	48.1
	No	174	51.9
The presence of soap and water	Yes	290	86.6
	No	45	13.4
The presence of hand washing sink	Yes	162	48.4
	No	173	51.6
The presence of wall mount/ individual ABHR	Yes	177	52.8
	No	158	47.2
The presence of gloves	Yes	266	79.4
	No	69	20.6
The presence of posters	Yes	137	40.9
	No	198	59.1
The presence of a protocol level	Yes	106	31.6
	No	229	68.4
Attitude	Positive	183	54,6
	Negative	152	45.4

### Factors associated with hand hygiene compliance

In bivariable logistic regression; knowledge of six steps of hand hygiene, knowledge on five moments of hand hygiene, having training for hand hygiene, presence of promotion for hand hygiene in the hospital, having adequate soap and water for hand hygiene, presence of adequate individual/wall matted alcohol-based hand rub for hand hygiene, presence of posters for hand hygiene, presence of protocol for hand hygiene, knowledge on hand hygiene compliance and attitude were found to be significantly associated variables with the hand hygiene compliance of health care providers.

After fitting these variables in multivariable logistic regressions; having trained for hand hygiene, have adequate soap & water for hand hygiene, presence of an adequate individual or wall matted alcohol-based hand rub, knowledge on hand hygiene compliance, and the attitude were significantly associated with the hand hygiene compliance. Study participants who have taken training about hand hygiene were 8.07 times more likely to have good hand hygiene compliance than those who have not taken training (AOR = 8.07, 95% CI: 2.91, 22.39). Study participants who have adequate soap and water for handwashing were 5.10 times more likely to have good hand hygiene compliance as compared to study participants who have not adequate soap and water for handwashing (AOR = 5.10, 95%CI: 1.93, 13.52). The odds of having good hand hygiene compliance were 3.23 times more likely who have alcohol-based hand rub individually/wall matted than those who did not have alcohol-based hand rub individually/wall matted. (AOR = 3.23, 95%CI: 1.32, 7.92). Those who were knowledgeable of hand hygiene were 6.74 times more likely to have good handwashing compliance than those who were not knowledgeable for hand hygiene (AOR = 6.74, 95%CI: 2.96, 15.34). Furthermore, Study participants who had a positive attitude towards hand hygiene were 2.15 times more likely to have good hand hygiene compliance as compared to who have a negative attitude for hand hygiene (AOR = 2.15, 95% CI: 1.04, 4.46) (Table 3**)**.

**Table 3:** Bivariable and multivariable logistic regression analysis of factors associated with hand hygiene compliance among health care providers in Central Gondar Zone public primary hospitals, Northwest Ethiopia, 2019 (*n* = 335)

Variables	Hand hygiene compliance			COR (95% CI)	AOR (95% CI)
		Good	Poor		
Knowing six steps of hand hygiene	Yes	43	202	2.52 (1.09, 5.84)	1.36 (.47, 3.91)
	No	7	83	1	1
Knowing five movements of hand hygiene	Yes	45	229	5.87 (1.38, 24.88)	3.5 (.16, 5.65)
	No	5	56	1	1
Trained for hand hygiene	Yes	45	165	6.55 (2.52, 16.98)	8.07 (2.91, 22.39)^a^
	No	5	120	1	1
Promotion for HH in Hospital	Yes	32	129	2.15 (1.15, 4.01)	1.57 (.74, 3.33)
	No	18	156	1	1
Have adequate soap & water for hand hygiene	Yes	44	190	3.67 (1.51, 8.91)	5.10(1.93, 13.52)^a^
	No	6	95	1	1
Presence of alcohol hand rub for individual	Yes	43	156	5.07 (2.21, 11.68)	3.23 (1.32, 7.92)^a^
	No	7	129	1	1
Presence of posters for hand hygiene	Yes	28	109	2.06 (1.12, 3.77)	.99 (.36, 2.67)
	No	22	176	1	1
Presence of protocol level for hand hygiene	Yes	26	80	2.78 (1.51, 5.12)	1.31 (.60, 2.85)
	No	24	205	1	1
Knowledge about hand hygiene	Good	41	140	4.71 (2.21, 10.07)	6.74(2.96, 15.34)^a^
	Poor	9	145	1	1
Attitude towards hand hygiene	Positive	34	149	1.94 (1.03, 3.67)	2.15 (1.04, 4.46)^a^
	Negative	16	136	1	1

^a^Statistically significant at α = 0.05, 1 = Reference group

## Discussion

Poor hand hygiene compliance of health care providers and its healthcare-associated infections have a greater impact on the patients in health care settings [13].

The present study demonstrated that overall good hand hygiene compliance was 14.9%. The finding the study was in line with other study conducted in University of Gondar teaching hospital, Ethiopia 16.5% [11] and lower than a studies conducted in Mali 21.8% [14], Kuwait 33.4% [15], India 43.4% [16] and black lion hospital, Ethiopia 79% [10]. But, this finding was higher as compared with a study carried out in Wachemo University teaching Hospital, Ethiopia 9.2% [17]. The variation might be due to study setting, sample size, a lack of awareness on healthcare-associated infections among health care providers, passive Infection Prevention and Control Committees (IPCCs) holding nonsystematic hand hygiene training and audits, inaccessibility of hand hygiene resources and it might be due availability of hand hygiene products and facilities. Alcoholic disinfectants were only used for disinfection of patients’ skin prior to aseptic procedures [18].

Knowledge of hand hygiene was associated with hand hygiene compliance. As a result, those who have good knowledge of HH had 6.74 times more compliance than those who have poor knowledge. This was in line with other similar study done in Kuwait which showed that knowledge of HCPs was significantly associated with good HH compliance [15]. The possible explanation might be due to knowledge on hand hygiene compliance will help to comply with hand hygiene with recommended way, knowledge will help to identify the advantage and disadvantages of hand hygiene compliance and identify the way of HCAIs transmission and how it is prevented.

Trained health care providers for hand hygiene were 8.07 times more likely to have good hand hygiene compliance than those who were not trained health care providers. Other studies were done in India [19], United Kingdom [20] and China [21] also showed that training had a positive relationship with HH compliance in all medical staff. This may be due to the fact that training built the knowledge of health care providers which had a significant association in HH compliance and those HCPs who had got training is expected to be a role model for others in terms of practicing good HH, Knowledge of HCPs will help to identify risk and benefits practice on the way of HCAIs transmission and how to prevent. A single lecture on basic hand hygiene protocols had a significant and sustained effect in enhancing hand hygiene compliance in a Swedish hospital [22]. A study conducted in University hospital in central Ethiopia showed compliance with hand hygiene at baseline and at follow up after training have a significant relationship with compliance [18].

The attitude was found to be significantly associated with HH compliance. As a result, those who had a positive attitude on hand hygiene had 2 times more compliance than a negative attitude. This was in line with other similar study done in Jordan which showed that the attitude of health care providers was significantly associated with good hand hygiene compliance [23]. Different reasons can be suggested for this, including the light workload they might have, the presence of Water Sanitation and Hygiene (WASH) committee, the presence of positive peer pressure, good professional attitude towards hand hygiene compliance, social factors, direct instruction from the respected body, personal experience, media and educational and religious institutions.

The odds of having good hand hygiene compliance were 3.23 times more likely who have alcohol-based hand rub individually/wall matted than those who did not get alcohol-based hand rub individually/wall matted. This is in line with other studies done in Taiwan [24] and Brazil [25]. The availability of alcohol-based hand rub resulted in significant improvement in hand hygiene compliance of health care providers. This might be due to the presence of alcohol-based hand rub the best way of improving hand hygiene compliance, the presence of alcohol-based hand rub at point of care was a reminder to health care providers to do hand hygiene and it might be easy for implementing hand hygiene. Inaccessibility of resources in their nearby ward might be one of the reasons for not practicing hand hygiene.

The other possible reasons are product selection on the antimicrobial profile, user acceptance, and cost. Additional activity against fungi (including molds), mycobacteria, and bacterial spores may be relevant in high-risk wards or during outbreaks [2] and they are often available as a gel, or on wipes. a study supports the fact that interactive educational programs combined with free availability of hand disinfectants significantly increased hand hygiene compliance [22]. Therefore, it is an excellent alternative to hand hygiene when antimicrobial efficacy, time for the procedure, and limited access to sinks are of concern [26].

The use of WHO advocated alcohol-based hand rubs is a practical solution to overcome these constraints because these can be distributed individually to staff for pocket carriage and placed at the point of care. The major advantage is that its use is well applicable to situations typical of developing countries, such as two patients sharing the same bed, or patient’s relatives being requested to help in care provision [14].

Health care providers who got adequate soap and water for handwashing were 5.10 times more likely to had good hand hygiene compliance as compared to health professionals who had not got adequate soap and water for handwashing. This is in line with the study done in Black Lion hospital, Ethiopia [10] but, there was no significant relation with adequate soap and water and hand hygiene compliance from a study conducted in Gondar university teaching hospital, Ethiopia [11]. This may be due to the difference in hospitals setting, availability, and accessibility of resources, user acceptance, and cost.

Like in other developing countries, the priority given to prevention and control of healthcare-associated infection is minimal. This is primarily due to lack of infrastructure, trained manpower, surveillance systems, poor sanitation, overcrowding and understaffing of hospitals, the unfavorable social background of population, lack of legislation mandating accreditation of hospitals and a general attitude of non-compliance amongst health care providers towards even basic procedures of infection control [8].

Improvement strategies of poor hand hygiene compliance among health care providers by ensuring that the necessary infrastructure and products are in place to allow hand hygiene performance at the point of care. This includes access to a safe, continuous water supply and the availability of soap and disposable towels, availability of effective and well-tolerated alcohol-based hand rub products at the point of care [2, 3, 27, 28].

Provision of regular training to all healthcare workers is essential to heighten awareness of microbial transmission by hands, emphasize the importance of hand hygiene and its indications, and to demonstrate the correct procedures for hand rubbing and hand washing. It may be achieved using regular presentations, e-learning modules, posters, focus groups, reflective discussion, videos, self-learning modules, practical demonstrations, feedback from assessment, or combinations of these and other methods and hand hygiene compliance health care providers may improve through placing reminders and prompts (posters, stickers, voice prompts, leaflets, gadgets, etc.) related to the importance of hand hygiene and the appropriate indications and procedures for its performance [29–36].

As far as a self-reported self-administered questionnaire the study may prone to social desirability bias. Since the study was done in public primary hospitals it may lack generalizability for private primary hospital.

## Conclusions

Hand hygiene compliance among health care providers in Central Gondar Zone public primary hospital was poor as compared to the WHO threshold. Training, attitude, the presence of alcohol-based hand rub in the working area, the presence of adequate soap and water in the working area were significantly associated with hand hygiene compliance. Implementing five movements of hand hygiene are the best method for preventing healthcare-associated infections. Therefore, the health care provider should be followed this principle to fight healthcare-associated infections.

## Data Availability

Data will be made available upon the reasonable request to the primary author.

## Abbreviations

AOR: Adjusted odds ratio
COR: Crude odds ratio
HCAI: Healthcare-associated Infections
HCP: Health care providers
HH: Hand hygiene
WHO: World Health Organization
